# Supplementary material for: Inflammatory profiles are associated with long COVID up to 6 months after COVID-19 onset: A prospective cohort study of individuals with mild to critical COVID-19
Source: PLoS One. 2024 Jul 15;19(7):e0304990. doi: 10.1371/journal.pone.0304990 (PMC11249251; doi:10.1371/journal.pone.0304990)

### **Supplementary Materials**

**Supplementary Table S1. Socio-demographic, clinical and COVID-19-related characteristics of RECoVERED participants included and excluded in the current analyses**

|  | **Total** | **Excluded** | **Included** | **p-value** |
| --- | --- | --- | --- | --- |
|  | **N=349** | **N=163** | **N=186** |  |
| Sex |  |  |  | 0.007 |
| Male | 196 (56%) | 79 (48%) | 117 (63%) |  |
| Female | 153 (44%) | 84 (52%) | 69 (37%) |  |
| Age, years | 51.0 (36.0-62.0) | 49.0 (33.0-61.0) | 52.0 (37.0-62.0) | 0.31 |
| BMI, kg/m^2^ | 26.2 (23.4-29.7) | 27.1 (23.5-30.7) | 25.7 (23.2-29.3) | 0.25 |
| BMI category |  |  |  | 0.092 |
| Normal weight | 141 (40%) | 66 (40%) | 75 (40%) |  |
| Overweight | 114 (33%) | 43 (26%) | 71 (38%) |  |
| Obese | 83 (24%) | 44 (27%) | 39 (21%) |  |
| Missing | 11 (3%) | 10 (6%) | 1 (1%) |  |
| Migration background |  |  |  | 0.033 |
| Dutch | 193 (55%) | 69 (42%) | 124 (67%) |  |
| Non-Dutch, OECD high-income | 40 (11%) | 18 (11%) | 22 (12%) |  |
| Non-Dutch, OECD low/middle income | 80 (23%) | 42 (26%) | 38 (20%) |  |
| Missing | 36 (10%) | 34 (21%) | 2 (1%) |  |
| Smoking |  |  |  | 0.47 |
| Non-smoker | 206 (59%) | 96 (59%) | 110 (59%) |  |
| Smoker | 22 (6%) | 10 (6%) | 12 (6%) |  |
| Ex-smoker | 102 (29%) | 40 (25%) | 62 (33%) |  |
| Missing | 19 (5%) | 17 (10%) | 2 (1%) |  |
| Highest level of education |  |  |  | 0.011 |
| None, primary or secondary education | 45 (13%) | 18 (11%) | 27 (15%) |  |
| Vocational training | 77 (22%) | 42 (26%) | 35 (19%) |  |
| University education | 185 (53%) | 64 (39%) | 121 (65%) |  |
| Missing | 42 (12%) | 39 (24%) | 3 (2%) |  |
| Number of COVID-19 high-risk comorbidities |  |  |  | 0.035 |
| 0 | 189 (54%) | 87 (53%) | 102 (55%) |  |
| 1 | 81 (23%) | 30 (18%) | 51 (27%) |  |
| 2 | 49 (14%) | 31 (19%) | 18 (10%) |  |
| 3 or more | 30 (9%) | 15 (9%) | 15 (8%) |  |
| Cardiovascular disease | 95 (28%) | 48 (31%) | 47 (25%) | 0.27 |
| Diabetes | 46 (13%) | 30 (19%) | 16 (9%) | 0.004 |
| Chronic respiratory disease | 26 (8%) | 14 (9%) | 12 (6%) | 0.39 |
| Cancer | 18 (5%) | 6 (4%) | 12 (6%) | 0.28 |
| Immunosuppressed | 6 (2%) | 2 (1%) | 4 (2%) | 0.54 |
| Psychiatric illness | 19 (6%) | 7 (4%) | 12 (6%) | 0.42 |
| Other comorbidities | 78 (23%) | 37 (24%) | 41 (22%) | 0.67 |
| Clinical severity score |  |  |  | 0.011 |
| Mild | 99 (28%) | 35 (21%) | 64 (34%) |  |
| Moderate | 151 (43%) | 72 (44%) | 79 (42%) |  |
| Severe/critical | 99 (28%) | 56 (34%) | 43 (23%) |  |
| Hospital admission | 179 (51%) | 105 (64%) | 74 (40%) | <0.001 |
| ICU admission | 45 (13%) | 19 (12%) | 26 (14%) | 0.63 |
| Days from illness onset to COVID-19 diagnosis | 4 (2-10) | 4 (2-11) | 5 (2-9) | 0.43 |
| Days from illness onset to hospitalisation | 9 (7-14) | 9 (6-14) | 9 (7-12) | 0.90 |
| Days from illness onset to ICU admission | 10 (7-12) | 9 (7-12) | 10 (7-11) | 0.71 |
| Received oxygen therapy before or during follow-up | 169 (49%) | 97 (61%) | 72 (39%) | <0.001 |
| Type of steroid |  |  |  | <0.001 |
| No steroid | 241 (69%) | 98 (60%) | 143 (77%) |  |
| Dexamethasone | 79 (23%) | 54 (33%) | 25 (13%) |  |
| Other steroid | 28 (8%) | 10 (6%) | 18 (10%) |  |
| Missing | 1 (0%) | 1 (1%) | 0 (0%) |  |
| Maximal HR, beats/min | 83 (72-94) | 87 (76-97) | 79 (71-92) | 0.002 |
| Maximal RR, breaths/min | 20 (16-24) | 20 (16-24) | 20 (16-22) | 0.050 |
| Lowest SpO_2_, % | 96 (91-98) | 95 (89-98) | 96 (92-98) | 0.040 |
| COVID-19 vaccination status (primary series) |  |  |  | <0.001 |
| Not vaccinated | 31 (9%) | 30 (18%) | 1 (1%) |  |
| Vaccinated | 228 (65%) | 43 (26%) | 185 (99%) |  |
| LTFU before vaccination | 90 (26%) | 90 (55%) | 0 (0%) |  |
| Time from illness onset to first vaccination, days | 244 (144-361) | 247 (77-345) | 244 (151-363) | 0.46 |
| Died during follow-up | 5 (1%) | 4 (2%) | 1 (1%) | 0.19 |
| Place of recruitment |  |  |  | <0.001 |
| Non-hospital | 161 (46%) | 55 (34%) | 106 (57%) |  |
| Hospital | 188 (54%) | 108 (66%) | 80 (43%) |  |
| Type of inclusion |  |  |  | 0.81 |
| Prospective | 257 (74%) | 121 (74%) | 136 (73%) |  |
| Retrospective | 92 (26%) | 42 (26%) | 50 (27%) |  |
| Days from illness onset to inclusion in study | 12 (6-38) | 14 (8-32) | 10 (5-62) | 0.14 |
| Prospective inclusions only | 9 (5-15) | 11 (7-16) | 8 (4-13) | <0.001 |
| Retrospective inclusions only | 85 (72-94) | 79 (52-94) | 87 (81-94) | 0.086 |
| Follow-up time from enrolment in study | 499.0 (274.0-659.0) | 274.0 (85.0-499.0) | 581.0 (486.0-696.0) | <0.001 |
| Lost to follow-up | 158 (45%) | 107 (66%) | 51 (27%) | NA |

**Supplementary Table S2**

|  | **Total** | **Recovered within 12 weeks (no PASC)** | **Did not recover within 12 weeks (PASC)** | **p-value*** |
| --- | --- | --- | --- | --- |
|  | **N=186** | **N=85** | **N=101** |  |
| Migration background |  |  |  | 0.33 |
| Dutch | 124 (67%) | 56 (66%) | 68 (67%) |  |
| Non-Dutch, OECD high-income | 22 (12%) | 13 (15%) | 9 (9%) |  |
| Non-Dutch, OECD low/middle income | 38 (20%) | 15 (18%) | 23 (23%) |  |
| Missing | 2 (1%) | 1 (1%) | 1 (1%) |  |
| Smoking |  |  |  | 0.34 |
| Non-smoker | 110 (59%) | 46 (54%) | 64 (63%) |  |
| Smoker | 12 (6%) | 5 (6%) | 7 (7%) |  |
| Ex-smoker | 62 (33%) | 33 (39%) | 29 (29%) |  |
| Missing | 2 (1%) | 1 (1%) | 1 (1%) |  |
|  | **Total** | **Recovered within 12 weeks (no PASC)** | **Did not recover within 12 weeks (PASC)** | **p-value** |
|  | **N=186** | **N=85** | **N=101** |  |
| Highest level of education |  |  |  | 0.002 |
| None, primary or secondary education | 27 (15%) | 7 (8%) | 20 (20%) |  |
| Vocational training | 35 (19%) | 10 (12%) | 25 (25%) |  |
| University education | 121 (65%) | 67 (79%) | 54 (53%) |  |
| Missing | 3 (2%) | 1 (1%) | 2 (2%) |  |
| Number of COVID-19 high-risk comorbidities |  |  |  | 0.17 |
| 0 | 102 (55%) | 51 (60%) | 51 (50%) |  |
| 1 | 51 (27%) | 24 (28%) | 27 (27%) |  |
| 2 | 18 (10%) | 4 (5%) | 14 (14%) |  |
| 3 or more | 15 (8%) | 6 (7%) | 9 (9%) |  |
| Cardiovascular disease | 47 (25%) | 17 (20%) | 30 (30%) | 0.13 |
| Diabetes | 16 (9%) | 7 (8%) | 9 (9%) | 0.87 |
| Chronic respiratory disease | 12 (6%) | 1 (1%) | 11 (11%) | 0.007 |
| Cancer | 12 (6%) | 6 (7%) | 6 (6%) | 0.76 |
| Immunosuppressed | 4 (2%) | 3 (4%) | 1 (1%) | 0.23 |
| Psychiatric illness | 12 (6%) | 7 (8%) | 5 (5%) | 0.36 |
| Other comorbidities | 41 (22%) | 15 (18%) | 26 (26%) | 0.18 |
| Symptom status at baseline |  |  |  |  |
| Symptomatic | 186 (100%) | 85 (100%) | 101 (100%) |  |
| Hospital admission | 74 (40%) | 19 (22%) | 55 (54%) | <0.001 |
| ICU admission | 26 (14%) | 6 (7%) | 20 (20%) | 0.018 |
| Days from illness onset to COVID-19 diagnosis | 5 (2-9) | 4 (2-7) | 6 (2-10) | 0.10 |
| Days from illness onset to hospitalisation | 9 (7-12) | 11 (8-15) | 8 (7-11) | 0.19 |
| Days from illness onset to ICU admission | 10 (7-11) | 10 (9-12) | 10 (7-11) | 0.69 |
| Received oxygen therapy before or during follow-up | 72 (39%) | 16 (19%) | 56 (55%) | <0.001 |
| Type of steroid received during COVID-19 |  |  |  | 0.045 |
| No steroid | 143 (77%) | 72 (85%) | 71 (70%) |  |
| Dexamethasone | 25 (13%) | 9 (11%) | 16 (16%) |  |
| Other steroid | 18 (10%) | 4 (5%) | 14 (14%) |  |
| Maximal HR, beats/min | 79 (71-92) | 77 (68-85) | 81 (72-97) | 0.012 |
| Maximal RR, breaths/min | 20 (16-22) | 18 (16-20) | 20 (16-25) | <0.001 |
| Lowest SpO2, % | 96 (92-98) | 97 (95-99) | 95 (90-98) | <0.001 |
|  | **Total** | **Recovered within 12 weeks (no PASC)** | **Did not recover within 12 weeks (PASC)** | **p-value** |
|  | **N=186** | **N=85** | **N=101** |  |
| Died during follow-up | 1 (1%) | 0 (0%) | 1 (1%) | NA |
| Place of recruitment |  |  |  | <0.001 |
| Non-hospital | 106 (57%) | 65 (76%) | 41 (41%) |  |
| Hospital | 80 (43%) | 20 (24%) | 60 (59%) |  |
| Lost to follow-up | 51 (27%) | 16 (19%) | 35 (35%) | NA |

**Supplementary Table S3. Concentration distribution of soluble inflammatory markers across different time-points among individuals with COVID-19 and health controls**

|  |  | **Persons with COVID-19** | | |  | **Healthy controls** | | |
| --- | --- | --- | --- | --- | --- | --- | --- | --- |
| **Marker** | **Weeks since symptom onset** | **Median** | **25th percentile** | **75th percentile** | ***p**** | **Median** | **25th percentile** | **75th percentile** |
| CRP  (log pg/ml) | ≤4 | 6.39 | 5.86 | 7.21 | 0.094 | 6.07 | 5.80 | 6.36 |
|  | 9-12 | 5.93 | 5.46 | 6.45 | 0.432 |  |  |  |
|  | 21-24 | 5.99 | 5.51 | 6.37 | 0.432 |  |  |  |
| sCD14  (log pg/ml) | ≤4 | 6.27 | 6.12 | 6.38 | p<0.001 | 6.72 | 6.34 | 6.95 |
|  | 9-12 | 6.13 | 6.01 | 6.21 | p<0.001 |  |  |  |
|  | 21-24 | 6.09 | 6.00 | 6.18 | p<0.001 |  |  |  |
| MCP1  (log pg/ml) | ≤4 | 2.73 | 2.59 | 2.87 | 0.351 | 2.66 | 2.60 | 2.75 |
|  | 9-12 | 2.65 | 2.54 | 2.80 | 0.48 |  |  |  |
|  | 21-24 | 2.60 | 2.51 | 2.77 | 0.202 |  |  |  |
| IP-10  (log pg/ml) | ≤4 | 1.83 | 1.60 | 2.05 | p<0.001 | 1.23 | 1.12 | 1.32 |
|  | 9-12 | 1.27 | 1.11 | 1.45 | 0.48 |  |  |  |
|  | 21-24 | 1.17 | 1.00 | 1.28 | 0.094 |  |  |  |
| IL-10  (log pg/ml) | ≤4 | 3.86 | 3.10 | 4.14 | p<0.001 | -1.00 | -1.00 | -1.00 |
|  | 9-12 | 3.00 | -1.00 | 3.10 | p<0.001 |  |  |  |
|  | 21-24 | -1.00 | -1.00 | -0.79 | 0.006 |  |  |  |
| IL-17  (log pg/ml) | ≤4 | 2.00 | 2.00 | 2.45 | p<0.001 | 0.00 | 0.00 | 0.00 |
|  | 9-12 | 2.00 | 0.70 | 2.00 | p<0.001 |  |  |  |
|  | 21-24 | 0.00 | 0.00 | 0.00 | 0.04 |  |  |  |
| sCD163  (log pg/ml) | ≤4 | 5.79 | 5.60 | 5.98 | 0.202 | 5.65 | 5.53 | 5.88 |
|  | 9-12 | 5.73 | 5.60 | 5.93 | 0.432 |  |  |  |
|  | 21-24 | 5.78 | 5.64 | 5.93 | 0.265 |  |  |  |
| IL-1𝛃  (log pg/ml) | ≤4 | 0.85 | 0.15 | 1.70 | p<0.001 | 0.15 | 0.15 | 0.15 |
|  | 9-12 | 0.15 | 0.15 | 1.11 | p<0.001 |  |  |  |
|  | 21-24 | 0.15 | 0.15 | 1.04 | p<0.001 |  |  |  |
| IL-6  (log pg/ml) | ≤4 | 3.38 | 2.55 | 4.32 | p<0.001 | 0.50 | 0.21 | 0.50 |
|  | 9-12 | 2.00 | 0.86 | 3.12 | p<0.001 |  |  |  |
|  | 21-24 | 0.59 | 0.08 | 1.60 | 0.047 |  |  |  |
| TNF-𝛂  (log pg/ml) | ≤4 | 3.21 | 2.88 | 3.69 | p<0.001 | 0.36 | 0.36 | 0.73 |
|  | 9-12 | 2.48 | 1.13 | 2.93 | p<0.001 |  |  |  |
|  | 21-24 | 0.57 | 0.06 | 1.03 | 0.283 |  |  |  |
| IL-2  (log pg/ml) | ≤4 | 0.30 | 0.30 | 0.54 | 0.202 | 0.30 | 0.30 | 0.30 |
|  | 9-12 | 0.30 | 0.30 | 0.30 | 0.005 |  |  |  |
|  | 21-24 | 0.30 | 0.30 | 0.30 | p<0.001 |  |  |  |
| IL-13  (log pg/ml) | ≤4 | 1.48 | 1.48 | 1.91 | 0.477 | 1.48 | 1.48 | 1.48 |
|  | 9-12 | 1.48 | 1.48 | 1.48 | 0.265 |  |  |  |
|  | 21-24 | 1.48 | 1.48 | 1.88 | 0.004 |  |  |  |

**Supplementary Figure S1**. **Log-concentrations of inflammatory markers over time since Illness onset among RECoVERED study participants, compared to healthy uninfected controls**


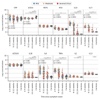


**Supplementary Figure S2. Correlation matrices of inflammatory markers at 0-4, 9-12 and 21-24 weeks after illness onset**


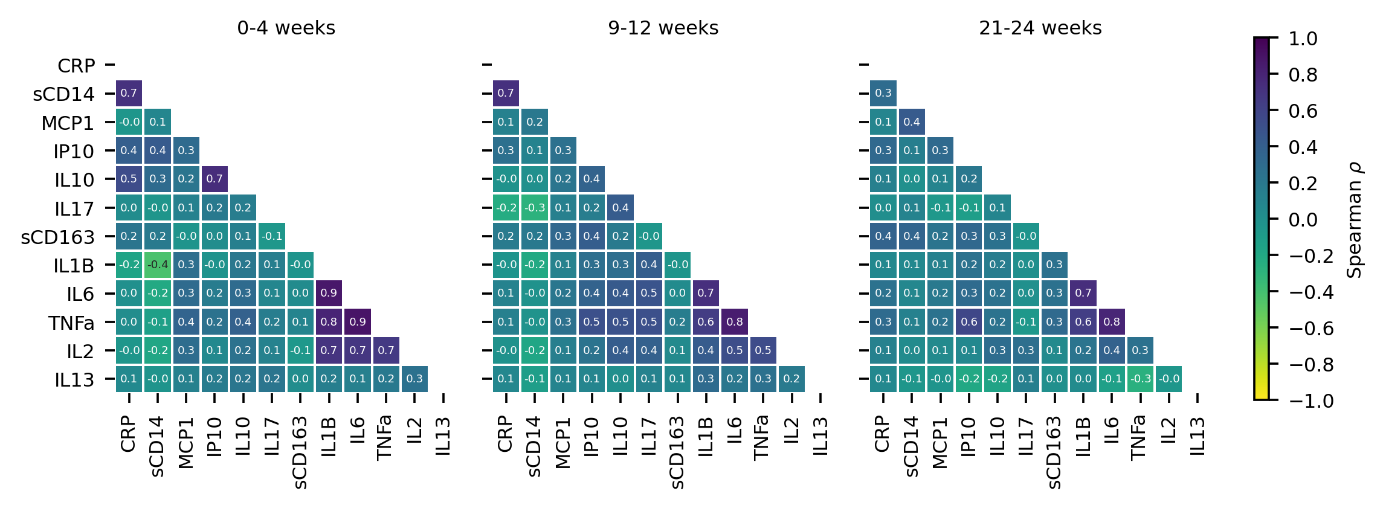


**Supplementary Figure S3. Log-concentrations of inflammatory markers at 9-12 and 21-24 weeks after Illness onset among RECoVERED study participants with initially mild or moderate COVID-19 only, stratified by PASC status**


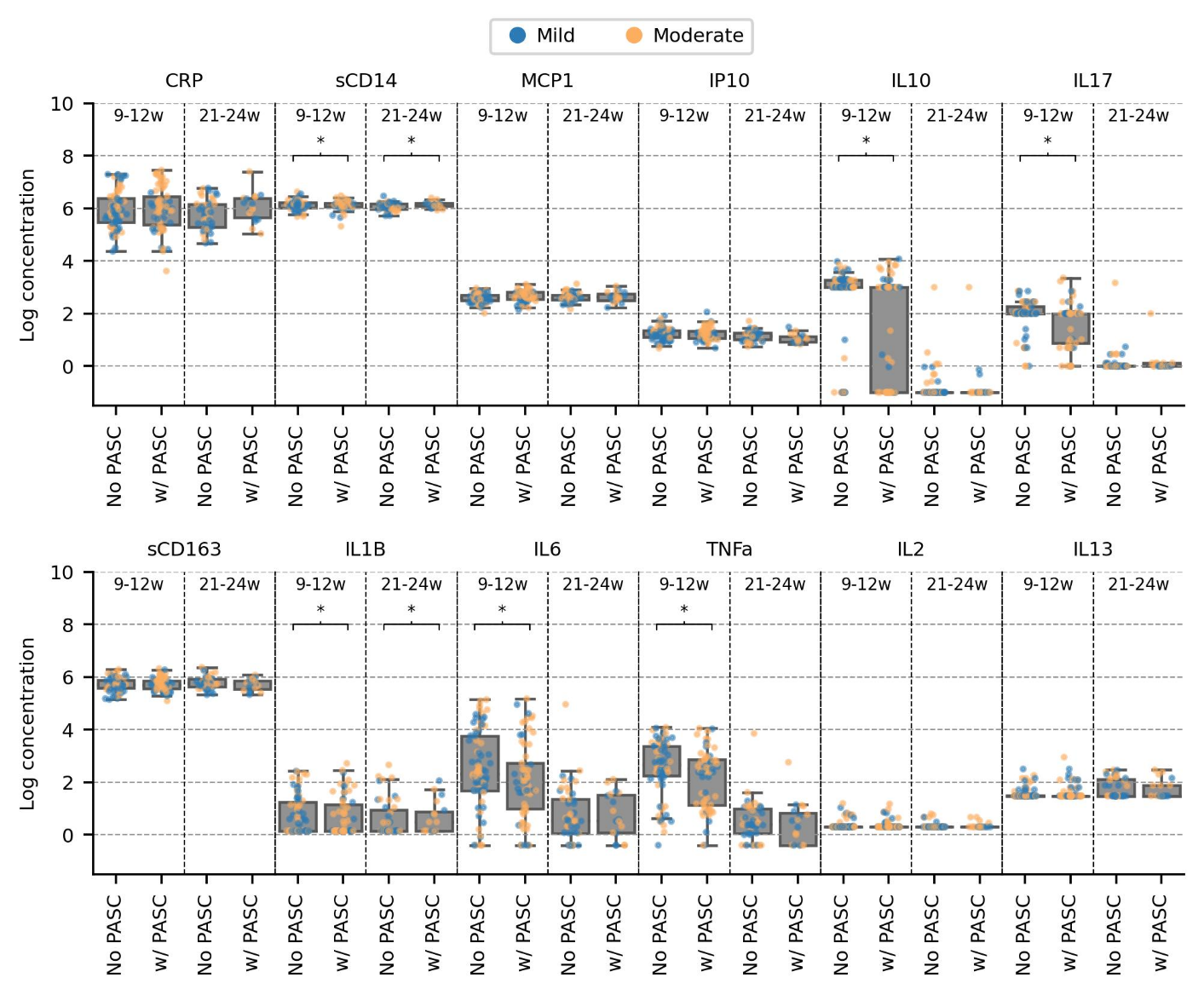


**Supplementary Figure S4. Log-concentrations of inflammatory markers at 21-24 weeks after illness onset among RECoVERED study participants, stratified by impaired diffusion capacity at month 6 after illness onset**

**
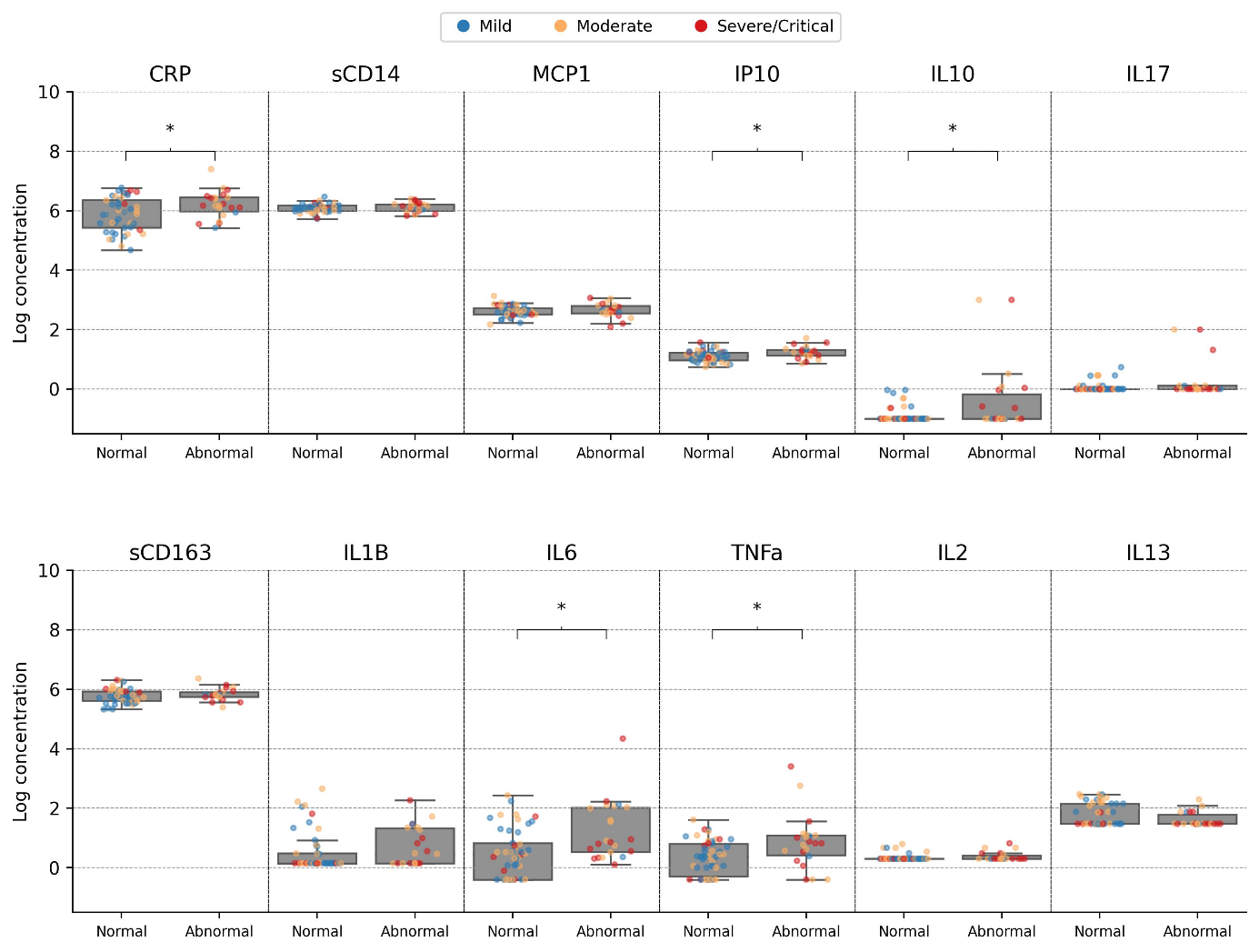
**

**Supplementary Figure S5. Multivariate linear regression analysis of factors associated with inflammatory marker concentrations at 21-24 weeks after COVID-19 illness onset, including abnormal lung function (impaired diffusion capacity)**


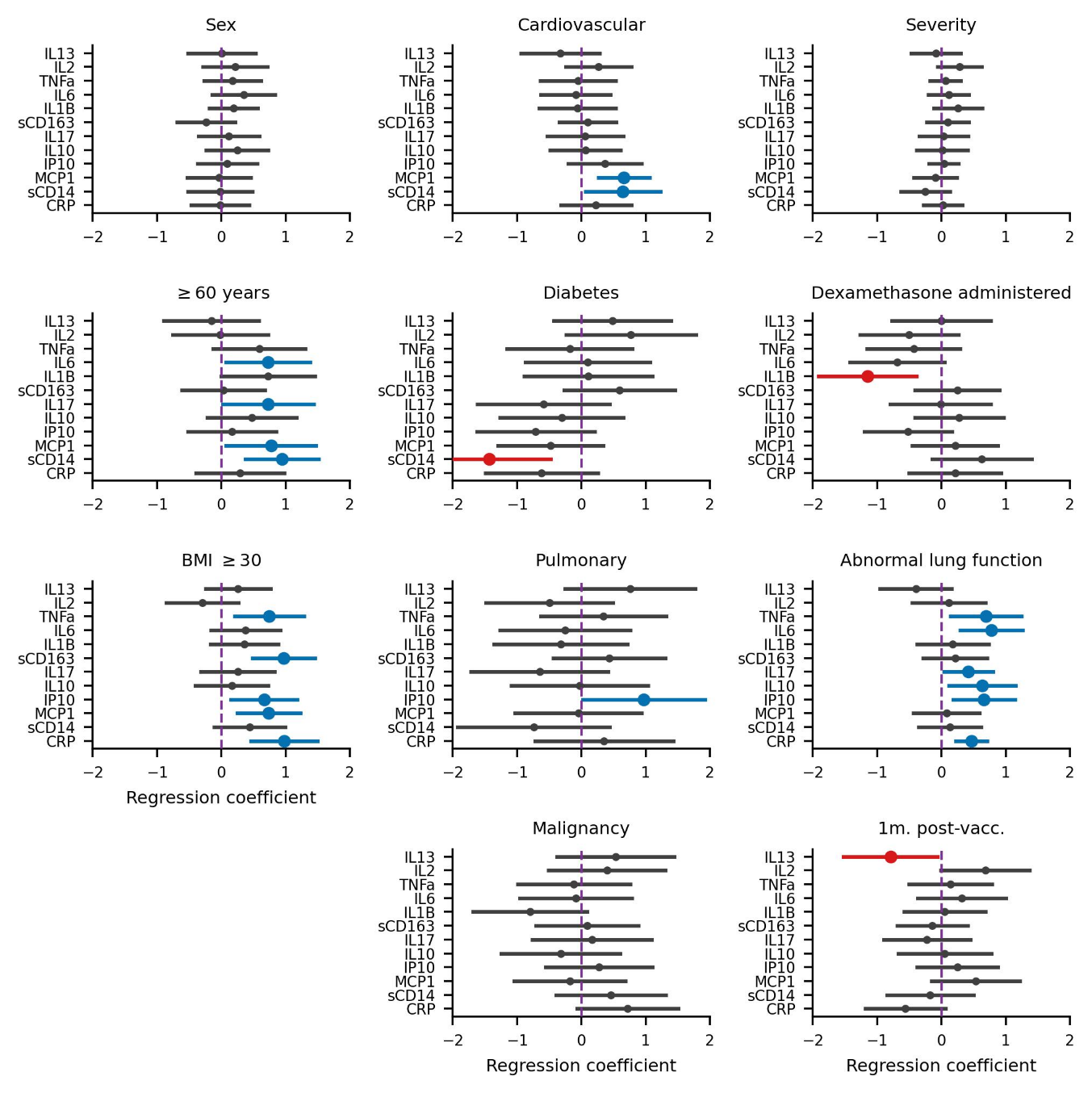

Supplement: S1 File — (DOCX) [file pone.0304990.s001.docx]
