# Supplementary material for: Inflammatory profiles are associated with long COVID up to 6 months after COVID-19 onset: A prospective cohort study of individuals with mild to critical COVID-19
Source: PLoS One. 2024 Jul 15;19(7):e0304990. doi: 10.1371/journal.pone.0304990 (PMC11249251; doi:10.1371/journal.pone.0304990)
Supplement: Attachment — Submitted filename: Response to the editor.docx [file pone.0304990.s002.docx]

**Response to the editor’s comments:**

*The title needs to be free of ambiguity: "Inflammatory profiles are associated with long COVID up to 6 months after mild to critical COVID-19: data from the RECOVERED cohort"*

| **Response** | **Revision made** | **Line numbers (in tracked changes document)** |
| --- | --- | --- |
| Thank you for your suggestion. We respectfully disagree that “data from the RECoVERED cohort” is less ambiguous than “a prospective cohort study of individuals with mild to critical COVID-19”, as the latter is more specific about the study population. We suggest that we add the name of the study to the title if the editor wishes for this to be included, bearing in mind that the title is now rather long. | Inflammatory profiles are associated with long COVID up to 6 months after illness onset: data from the prospective RECoVERED cohort of individuals with mild to critical COVID-19 | Title |

*Introduction: the second paragraph needs to be reduced. I would mention the current pathophysiologic hypotheses for long COVID (viral persistence, chronic inflammation, hypercoagulability and autonomic dysfunction: see https://doi.org/10.1038/s41577-023-00966-7) and then briefly describe what is known and unknown about the chronic inflammation theory (because your study dealt with the chronic inflammation theory). The third paragraph also needs to be reduced. You could say something like: "The aim of our study was to further explore the chronic inflammation theory about the pathophysiology of long COVID through a longitudinal assessment of inflammatory cytokines present in individuals with COVID-19 at disease onset."*

| **Response** | **Revision made** | **Line numbers (in tracked changes document)** |
| --- | --- | --- |
| Thank you for your suggestion. We shortened the second and third paragraphs of the discussion by leaving out in-depth discussion of specific papers. We have outlined and referenced wider literature of what the “chronic inflammation theory” entails. We did not include the statement suggested of “cytokines present in individuals with COVID-19 at disease onset” as this is not wholly accurate, as initial serum samples were taken <4 weeks of illness onset. However, we hope that you agree that our aims are stated in a clear and unambiguous way.  Please note that the article referenced in the editor’s comment is under paywall. | Paragraphs shortened. | 116-151 |

*Page 7 line 100: it will be helpful for the readers to see the reference for STROBE guidelines.*

*Line 103: before describing RECOVERED, you would like to state something like: "This is a subset of the RECOVERED study."*

| **Response** | **Revision made** | **Line numbers (in tracked changes document)** |
| --- | --- | --- |
| Thank you for your suggestions. These changes have been implemented. | Reference added and statement about sub-set of study population added. | Lines 154 and 166 |

*"We understand that a COVID-negative control group is not necessary to assess the impact of PASC on cytokine concentrations. However, for clinical relevance it is not only useful to know the relative*

*effect of PASC on cytokine concentrations but also whether the levels reported are of clinical significance. This can only be determined by observing cytokine levels in an uninfected, healthy reference population." I still do not see the importance of that group. You start the study already knowing the physiologic and abnormal levels of the serum cytokines you assessed in the study. You only need to mention the healthy individuals as laboratory controls when reporting about laboratory assay of serum cytokines. THERE IS NO CONTROL GROUP IN A COHORT STUDY. THE COHORT STUDY HAS TWO STUDY POPULATIONS: THOSE WHO GO ON TO DEVELOP THE DISEASE OF INTEREST (LONG COVID FOR THIS STUDY) AFTER CONTACT WITH THE EXPOSURE (COVID-19 FOR THIS STUDY), AND THOSE WHO DO NOT DEVELOP THE DISEASE (FOR THIS STUDY: THOSE WHO HAD covid-19 AND DID NOT DEVELOP LONG COVID).*

| **Response** | **Revision made** | **Line numbers (in tracked changes document)** |
| --- | --- | --- |
| Thank you for your suggestions. We would like to point out that most of the cytokines explored in our study are actually not routinely assessed in clinical practice and therefore absolute cut-offs are not well described. Therefore, having a reference value from a healthy, uninfected population allows us to assess the effect of SARS-CoV-2 infection and comorbidities on cytokine levels as well as the effect of PASC. This facilitates a more robust understanding of the possible confounding factors in our analyses that could be associated with aberrant cytokine levels. We agree that this is a reference and not a control group and have adjusted the wording in the manuscript.  We agree with your description of the exposure and outcomes in our study. | New text: “We additionally included reference u samples from SARS-CoV-2-uninfected, healthy individuals (i.e., no comorbidities) collected between March 2020 and November 2021, **in order to better understand the impact of SARS-CoV-2 infection on absolute levels of cytokines**.”  (in **bold** added text) | Lines 170-171 |

*After the sub-section "Study design and participant enrolment", please mention a section " Data collection " under which you detail the clinical and laboratory (do not forget to specify the cytokines assessed [ideally classified by families such as IFN-related cytokines, chemokines, TNF cytokines, interleukins: I am surprised I do not see IL-6 a very important pro-inflammatory cytokine on line 140] and say why you assessed specifically those cytokines since there are many pro-inflammatory cytokines; CD14 and CD163 are monocyte/macrophage surface markers, not cytokines I therefore do not understand why they are reported and CRP on line 138) data collected, the methods of data collection used, and the timings of data collection (to, at 3 months, at 6 months). So, this sub-section will contain information from the line 119 to the line 145.*

| **Response** | **Revision made** | **Line numbers (in tracked changes document)** |
| --- | --- | --- |
| Thank you. We have made the suggested changes. Socio-demographic and symptom data collection is now under the same heading as serum sampling and cytokine assays, as requested. | “C-reactive protein (CRP), monocyte/macrophage surface receptors (CD14, CD163), tumor necrosis factor (TNF)-α, interferon-γ-inducible protein 10 (IP-10)/CXCL10, monocyte chemoattractant protein (MCP)1/CCL2, and interleukins (IL)1β IL2, IL6, IL10, IL13 and IL17A, concentrations were analyzed in serum by human magnetic luminex screening assay (LXSAHM-02 and LXSAHM-10; R&D Systems). using the Bio-Plex 200 System (Bio-Rad Laboratories).” | 194-198 |

The title states that the total duration of the follow up for each participant was six months, so I do not understand why on line 131 you mention that you collected blood samples at 12, 18 , 24 months.

| **Response** | **Revision made** | **Line numbers (in tracked changes document)** |
| --- | --- | --- |
| Thank you for this suggestion. We have made the requested change. | “Serum samples were collected at day 0 and 7 and subsequently at months 1, 3, and 6, of follow-up.” | 186-187 |

*After the data collection sub-section, add a sub-section termed "Outcome" which is very important for a cohort. The main outcome here being the serum levels of pro-inflammatory cytokines at 6 months post-COVID infection. After the outcome sub-section, then follow the definitions and statistical analysis sub-sections as you did.*

| **Response** | **Revision made** | **Line numbers (in tracked changes document)** |
| --- | --- | --- |
| Thank you for this suggestion. We agree that it will be clearer for the reader to have a separate heading under which outcomes are listed. | Outcomes  The primary outcomes of our analysis were cytokine levels at 9-12 and 21-24 weeks after COVID-19 illness onset. Our secondary outcomes, when assessing possible early biomarkers for later PASC, were: PASC status at 6 months, and CRP and IL-6 levels at 21-24 weeks. | 204-207 |

*Line 153 "COVID-19 clinical severity was categorised according to WHO criteria[20]". Please, describe those grades of severity.*

| **Response** | **Revision made** | **Line numbers (in tracked changes document)** |
| --- | --- | --- |
| Thank you for this advice. We had initially chosen not to list the full definitions to ensure we did not exceed the suggested word count but now have added this information to the manuscript. | COVID-19 clinical severity was categorised according to WHO criteria [14]: mild disease was defined as having a RR <20/minute and SpO2>94% on room air at both D0 and D7 study visits; moderate disease as having a RR20–30/minute and SpO2 90–94% on room air (or receiving oxygen therapy, if no off-oxygen measurement available) at either visit; severe disease as having a RR >30/minute and SpO2<90% on room air (or receiving oxygen therapy) at either visit; critical disease as requiring ICU admission as a result of COVID-19 at any point.. | 214-220 |

*Lines 154-156: "Diffusion capacity (DLCO) at 6 months after illness onset was defined as impaired according to American Thoracic Society (ATS) European Respiratory Society guidelines[21], as described previously described[19]". Is this not a secondary outcome of the study? If it is the case, then do not forget to mention that in the outcome sub-section.*

| **Response** | **Revision made** | **Line numbers (in tracked changes document)** |
| --- | --- | --- |
| The DLCO was used as an alternative exposure variable (instead of PASC). Therefore, we have not included it in the outcome section. | N/A | N/A |

*Lines 159-161: "Socio-demographic, clinical and study characteristics were compared between included participants with and without PASC at 12 weeks after illness onset. To assess selection bias, we compared features of included and excluded participants." Please, delete.*

| **Response** | **Revision made** | **Line numbers (in tracked changes document)** |
| --- | --- | --- |
| In our opinion it is important to consider selection bias, therefore we decided to keep the descriptive analyses and assessment of selection bias of the sub-set of participants of the RECoVERED study included in this paper. | None | N/A |

*Generally speaking, your statistical analysis sub-section is long, difficult to read and confusing. Please, state the software where you stored the data collected, whether you needed to export data to another software for statistical analysis, the statistical tests used for the different study outcomes, the statistical tests used to assess the factors associated with the study outcomes, the candidate factors associated with the different outcomes as assessed in the statistical models, and the definition of statistical significance you used.*

| **Response** | **Revision made** | **Line numbers (in tracked changes document)** |
| --- | --- | --- |
| We restructured the statistics section according to the editor’s suggestions. | See manuscript | 235-281 |

*A cohort based on STROBE should start with the sub-section " Baseline characteristics of the study population", Then follow the sub-sections "primary outcome(s)" and "secondary outcomes". Then you can end the results section with the sub-section " factors associated with persistence of a pro-inflammatory profile in individuals with COVID-19".*

| **Response** | **Revision made** | **Line numbers (in tracked changes document)** |
| --- | --- | --- |
| Thank you, we have implemented these changes to the structure of the Results section. | See manuscript |  |

Discussion: last time, I proposed a discussion plan. Please, stick to it. The paragraph about study's strengths and limitations should be shortened: regarding limitations, please stick to comments about the shortcomings of a 6-month prospective cohort study.

| **Response** | **Revision made** | **Line numbers (in tracked changes document)** |
| --- | --- | --- |
| As suggested, we have shortened the limitation section. | See manuscript | 454-477 |

*Line 380 "In summary" needs to be deleted. The conclusion should match with the main objective stated in the introduction, and call for further studies on the topic of your study.*

| **Response** | **Revision made** | **Line numbers (in tracked changes document)** |
| --- | --- | --- |
| Thank you, we have deleted this phrase and ensured the conclusion is in line with the main aims of the study. | Our study indicates that immune dysregulation is associated with PASC, especially when defining PASC as having impaired pulmonary function. In addition, early raised IL1β levels were strongly predictive of ongoing PASC at 6 months in our analyses. Our findings therefore suggest that immune dysregulation plays an important role in the pathogenesis of ongoing symptoms. | 480-487 |

*Ultimately, the abstract will also need a revision, and should not exceed 300 words. The keywords should contain terms from the title and eventually the abstract.*

| **Response** | **Revision made** | **Line numbers (in tracked changes document)** |
| --- | --- | --- |
| We are unsure which revision of the abstract would be needed. The current abstract is 248 words (not exceeding 300 words) and we checked the the key words for matching words in title and abstract. | None | N/A |
