# Supplementary material for: Inflammatory profiles are associated with long COVID up to 6 months after COVID-19 onset: A prospective cohort study of individuals with mild to critical COVID-19
Source: PLoS One. 2024 Jul 15;19(7):e0304990. doi: 10.1371/journal.pone.0304990 (PMC11249251; doi:10.1371/journal.pone.0304990)
Supplement: Attachment — Submitted filename: Response to Reviewers.docx [file pone.0304990.s003.docx]

Reviewer’s / editorial comments:

The manuscript has been improved, but there are some revisions that need to be made so that the published report matches the level of field work made.

*Ref 16 should be revised:* **it is unclear how this reference should be revised, this reference refers to the STROBE guideline and checklist that is available on the mentioned website.**

*Tables should come at the end of the full-text manuscript, to facilitate the assessment of the manuscript:* **following this comment, table 1 is moved to the end of the manuscript, see p23**

*Lines 100-102: the citation of the WHO is needed:* **this reference was added**

*Line 136: the reference provided is not right. Is there a link with reference 18 cited in line 160?* **indeed reference 1 is incorrect: the right reference is indeed no 18: this is changed accordingly**

*Lines 153 and 158: 21 or 20?* **We believe the editor is referring to the number of COVID-19 symptoms included in our analyses. The editor is correct that the total count is 21 symptoms, not 20, and this has been changed in the manuscript accordingly.**

*I advise against citing preprints. If you have to cite a preprint, specify that the reference is a preprint:* **we checked the manuscript for references to pre-prints and updated this (ref 15) to the publication**

*Line 175: consider writing "non-specific antinuclear antibodies" instead of "total antinuclear antibodies":* **at this point we disagree with the reviewer: antinuclear antibodies encompass specific antibodies (for instance adsDNA) and therefore cannot be named “non-specific”.**

*Line 176: please rephrase "as indicators of possible auto-immunity" as "as markers of autoimmunity". Indeed, ANA are the best markers of autoimmunity*: **this was changed accordingly.**

*Lines 182-183. I suggesting deleting "when assessing possible early biomarkers for later PASC" as it brings confusion to the message you want to give:* **this was changed accordingly.**

*Line 191: ref [14] is not the WHO reference.* **this was changed accordingly.**

*Line 198 American Thoracic Society European Respiratory Society should read American Thoracic Society/European Respiratory Society:* **this was changed accordingly.**

*Line 199: "described" should be deleted:* **this was changed accordingly.**

*Lines 204 and 205: "To assess selection bias, we compared features of included and excluded participants." This sentence is not right and should be deleted. In fact, all the paragraph in lines 203-207 is not necessary:* **in our opinion it is relevant to assess selection bias as our analyses were performed on a sub-set of our total cohort, and therefore we decided to leave this section in (of note selection bias data are presented in table S1 and in the first section of the results paragraph). It is important for the reader to know, for instance, that samples from male participants were more likely to be included in our analyses than from female participants, as it affects the external validity of the findings.**

*The paragraph in lines 233-240 is difficult to understand. Are the early predictors of PASC you are talking about different from the pre-COVID and COVID-related factors you are talking about in the paragraph in lines 219-231?* **With earlier predictors of PASC we mean predicting factors at illness onset. This was clarified in the text (see l239).**

*You say that you have measured the levels of non-specific ANA within 4 weeks of COVID onset. However, I do not see how you used that data in the predictor analysis:* **please refer to results section lines 344- 346 and figure 4A: here we list the data on ANA as predictive factor for PASC.**

*When describing the statistical analysis, focus on the description of: 1) The statistical tests used and specify the variables that were analysed using those statistical tests. Here again, focus on the study outcomes so that readers will be able to have similar results using those statistical tests. This is why I said that the paragraph in lines 203-207 is not necessary. 2) The definition of statistical significance for the outcomes of interest, 3) then how you reported the results. 4) finally, the software where you stored the data before the analysis and how you exported the data from that software to the statistical analysis software. It reads something like this:*

*"We determined box and whisker plots of serum concentrations of cytokines over time. During this analysis, data of study participants with and without PASC at 12 and 24 weeks after COVID-onset were compared with data from the uninfected healthy control population using the Mann-Whitney and Bonferroni correction tests. We segregated serum cytokine levels at 21-24 weeks post-COVID by DLCO status (normal vs abnormal). We subsequently used the linear mixed-effects test to assess the effect of pre-COVID and COVID-related factors on serum concentrations of cytokines at 12 and 24 weeks post-COVID. The pre-COVID factors assessed included.... [reference important to show that you chose them based on the literature]. The COVID-related factors assessed included ....[reference again]. We used a correlation matrix to help assess the reciprocal associations between cytokines at 9-12 and 21-24 weeks post-COVID. Add a comprehensive synthesis of the paragraph in lines 233-240 here if this paragraph is still necessary. All data were collected and stored in a secure database and exported for statistical analyses to Python. Analyses were performed using the statsmodels package (v. 0.13.2)[21], whilst the random forest regression analyses were performed in Python using the scikit-learn package (v.2441.1.3).":* **in our opinion the statistics paragraph does explain in a logical order the tests used and is displayed in analogy to the results section. However, we added the suggestions of the reviewer and also more clearly highlighted which sections belong to the different study outcomes.**

*Paragraph in lines 248-255: state the specific numbers, avoid the adverbs:* **I am unsure what the reviewer means, in this section we do list the numbers and I do not see irrelevant adverbs.**

*Table 1 is confusing and needs to be revised because it is supposed to display the baseline characteristics, but I see the 3-month data in that table:* **this may be a misunderstanding: table 1 does show the baseline characteristics (second column) and the baseline characteristics of groups stratified for participants that later in the study develop PASC (third and fourth column). To clarify this information was added to the legend of the table.**

*Follow-up data should not be in that table:* **please refer to our previous answer**

*I also do not understand why there is a word recovered in that table whereas study participants belong to the RECOVERED cohort. This may be confusing for readers: it is difficult to find a proper synonym for recovered (from illness symptoms).* **We changed it for now in resolved symptoms and ongoing symptoms.**

*The table is unacceptably long. A shorter title such as "Baseline characteristics of study participants with and without PASC after COVID-19 infection" would be better:* **this was changed accordingly**

*Then, you see that the relevant comparisons is that between the two study populations reported in the table's title. If you want to show the baseline characteristics of the other study population, do this in tables that you send to the supplementary material. Variables in table 1 should be those that you found relevant predictors of PASC, along with demographic characteristics: according to the comment the table 1 was shortened and the following data:* **sex, BMI, initial disease severity, vaccination and demographic predictors were included. All other baseline data that we consider relevant in order to have insight in the study population characteristics were moved to a new supplementary table (s2).**

*I would organize its arrows like this: demographic (age, sex, level of education) and epidemiological (e.g., COVID-19 vaccination status, comorbidities) data, followed by clinical data (symptomatic/asymptomatic at diagnosis, cardiorespiratory features, disease severity: mild, moderate, critical), and then management data (ambulatory/hospital/ICU care, drugs used: steroids, other immunomodulatory and immunosuppressive drugs). I would not mention the migration status. You can also merge all the comorbidities in a unique variable "comorbidities":* **since the data displayed in the table are now re-arranged (see previous answer) some of the here mentioned factors are now in suppl table 2.**

*I am happy to see the vaccination status in table 1, but I do not see it among the candidate predictors of PASC in the methods section. Please, make a comment about this in the statistical analysis sub-section of the Methods section:* **vaccination status is already mentioned in the statistics section: see lines 230 and 231.**

*I suggest deleting the text in lines 274-276 which may be confusing. It is already clear in the methods that you used reference samples in the laboratory when assessing serum levels of cytokines:* **we suggest to leave this in since the text here (in the current revised manuscript (v3) l261-263) lists the characteristics of the reference samples which belong in the results section.**

*Lines 277-363: there should be consistency between the way you report these results and the report on study methods. For example, I see "in univariate analysis", "in multivariate analysis", but I have not seen that in the statistical analysis sub-section*: **the method used for the multivariate analysis in the results section is the linear mixed effect model as described in the methods / statistics section. We clarified this in the revised version of the statistics section.**

*You report that determinants of serum cytokine levels are part of the primary outcome, but this is not what I have read in the methods section:* **this was added to the methods section (l212).**

*There is this problem of whether you make a difference between early predictors of PASC and pre-COVID and COVID-related factors highlighted above that is also apparent here. Instead of saying after "illness onset", state "after COVID-19 onset" throughout the text:* **accordingly, illness onset is replaced by COVID-19 onset throughout the text of the manuscript.**

*Focus this report of results on data from participants with and without PASC after COVID-19 please. No need to emphasize on the population from which you collected reference samples. When you state in the text that this result is available in supplementary tables...Readers will themselves go and find in those tables results of the population from which you collected reference samples. This is the most crucial part of the result section. Write it in the simplest and most intelligible way referring readers to the figures that are very beautiful Draw our attention to the figures as much as you can:* **we thank the reviewer for this compliment**

*A more exact name of Figure 1 is "Box and Whisker plots of serum cytokine levels in the study population at 9-12 and 21-24 weeks post-COVID":* **the title was changed accordingly**

*I can not assess the other figures until you resolve the inconsistency across the methods section and lines 277-363 of the results section:* **please refer to our answer at the top of page 2.**

*Lines 366-367: do you want to say that you assessed the evolution of cytokines in individuals with PASC up to 6 months (24 weeks) post-COVID?:* **indeed, that was also stated as such in the abstract: “We described longitudinal trajectories of cytokines in adults up to 6 months following SARS-CoV-2 infection and explored early predictors of PASC”.**

*The study objective, methods, results and discussion should be consistent. Please, take your time to fix these issues before submitting the revised version of the manuscript. You have strong results. Strengthen your discussion. The first paragraph of the discussion should summarize the study results in a way that is consistent with the objective stated in the introduction, as well as methods and results sections:* **we re-read this first paragraph and checked the consistence with the abstract as well as our primary and secondary aims and feel that as the text is now there is consistence throughout the manuscript.**  **The reviewer suggests to “strengthen the discussion”, if that is indeed needed then specific suggestions would be welcome.**

*The second paragraph should explain the results of Figure 1 taking into consideration data from the literature, and you know that this is an ever-evolving field. So, try as much as possible to have the latest evidence when making this discussion:* **following this comment a few recent landmark papers were added as reference (Peluso Semin Imm 2024; Yin Nat Imm 2023; Thaweethai JAMA 2023).**

*In the subsequent paragraph, explain your results on predictors of high levels of pro-inflammatory serum cytokines and how those predictors and the pro-inflammatory cytokines could influence the pathogenesis of PASC:* **we added this to the discussion (lines 492-498).**

*Then discuss the implications of your research on this currently very famous research area. How do you advance our knowledge? What remains to be done to further elucidate the hypotheses you elaborated while interpreting your results:* **this was added to the final part of the conclusion (lines 528-532)**

*Finally, state the limitations and strengths of your study:* **We think the reviewer has overlooked the limitation as listed in the discussion see lines 508-520**

*I am surprised that you measure non-specific ANA, and you did not report the titers of ANA in your study population (I would put them among clinical data in table 1. COVID-19 is a confirmed risk factor of autoimmune diseases. It is not clear whether autoimmunity is involved in the pathogenesis of PASC. I thought that may be you will provide an insight into the involvement of autoimmunity in the pathogenesis of PASc, especially since you know that interferons (e.g. IP- 10) are involved in the pathogenesis of autoimmune diseases and autoantibody production s a downstream reaction of interferon signaling:* **we agree with the reviewer that there are several studies that show a potential role for auto-immunity in development of PASC. There are however different categories of autoimmunity that each have a different pathophysiological background (Bodansky JCI Insight 2023, Peluso CID 2022, ). ANA abs for instance are of a different category than antibodies against type 1 IFNs or cytokines. It was beyond the scope of our study to generate a comprehensive analysis of all types of auto-antibodies and we only included ANA abs. Following the suggestion, we added ANA data to table 1.**

*It is in the conclusion that it becomes very clear that you defined PASC as impaired DLCo in some circumstances. This is not so clear in the methods (lines 186-199) and results section, unfortunately. Clearly state in the methods section:* **the suggestion that we define PASC by impaired DLCo seems a misinterpretation of the reviewer. We define PASC based on self-reported symptoms (l 159 ev “PASC was defined as reporting at least one COVID-19 symptom that, from COVID-19 onset, occurred within one month and continued beyond 12 weeks”). The section lines 383-385 in the results section may have been confusing: “when substituting PASC status for impaired diffusion capacity, we found that impaired diffusion capacity was associated with higher levels of CRP, IL6, TNFα, IP10, IL10 and IL17 in multivariable analyses.”. For clarification we reformulated this sentence and omitted PASC status (see adjustment in results l383). Finally, we reformulated lines 514-516 in the discussion to better align with our previous explanation of the approach taken.**

*The conclusion should be strong. What message would you want to remember if you forgot everything about your manuscript? Put that message in the conclusion. I guess it will fit with the study's main objective:* **see adjustment in the conclusion (l534-538).**

Format references according to the PLOS ONE policy: **the references in text and reference list were adjusted.**
